# Induction of Tolerance and Immunity by Dendritic Cells: Mechanisms and Clinical Applications

**DOI:** 10.3389/fimmu.2019.02393

**Published:** 2019-10-29

**Authors:** Jitka Fucikova, Lenka Palova-Jelinkova, Jirina Bartunkova, Radek Spisek

**Affiliations:** ^1^Sotio, Prague, Czechia; ^2^Department of Immunology, 2nd Faculty of Medicine and University Hospital Motol, Charles University, Prague, Czechia

**Keywords:** dendritic cells, immunotherapy, cancer, autoimmune disorders, vaccine preparation

## Abstract

Dendritic cells (DCs) are key regulators of immune responses that operate at the interface between innate and adaptive immunity, and defects in DC functions contribute to the pathogenesis of a variety of disorders. For instance, cancer evolves in the context of limited DC activity, and some autoimmune diseases are initiated by DC-dependent antigen presentation. Thus, correcting aberrant DC functions stands out as a promising therapeutic paradigm for a variety of diseases, as demonstrated by an abundant preclinical and clinical literature accumulating over the past two decades. However, the therapeutic potential of DC-targeting approaches remains to be fully exploited in the clinic. Here, we discuss the unique features of DCs that underlie the high therapeutic potential of DC-targeting strategies and critically analyze the obstacles that have prevented the full realization of this promising paradigm.

## Introduction

Immune responses result from a complex interplay between the innate and adaptive immune system. Dendritic cells (DCs) are an important subset of antigen-presenting cells (APCs) that specialize in priming different types of effector T cells and, thus, tailor the outcome of an immune response and having a central role in the immune system with a unique ability to control both immunity and tolerance. Compared to other APCs such as macrophages and B cells, DCs are considered the most efficient APCs capable of efficiently processing and presenting exogenous antigens on both MHCII and MHC I molecules to naïve CD4^+^ and CD8^+^ T cells, respectively, thus initiating the adaptive immune response. DCs were first discovered in 1973 by Ralph Steinman, who was awarded a Nobel Prize in 2011 for that discovery. DCs comprise a heterogeneous population of bone-marrow-derived cells that are seeded in all tissues. Five major types of DCs can be distinguished: plasmacytoid DC (pDCs), type 1 conventional DCs (cDC1), type 2 cDCs (cDC2) also referred to as myeloid DCs (mDCs), Langerhans cells and monocyte-derived DCs (MoDCs) (1–4), which differ in their phenotype, localization, and function as summarized in Table 1 (2, 5–7). In peripheral tissues, DCs capture antigens using different mechanisms. DCs loaded with antigens subsequently migrate into the draining lymph nodes via afferent lymphatics, where peptides loaded on DCs histocompatibility complex (MHC) class I and II molecules to be recognized by T-cell receptor (TCR) on T lymphocytes (8). Immature DCs (iDCs) can present self-antigens to T cells to maintain immunological tolerance either through T cell deletion, induction of T cell anergy or the differentiation of regulatory CD4^+^CD25^+^FoxP3^+^ T cells (Tregs) (9). After encountering appropriate stimuli, DCs differentiate into mature DCs, which are characterized by a decrease in endocytic activity, upregulation of MHC class I and II molecules and costimulatory molecules and responsiveness to inflammatory chemokines (10). Mature, antigen-loaded DCs promote the differentiation and activation of T cells into effector T cells with unique functions and cytokines profiles by providing immunomodulatory signals through cell-cell contacts and cytokines (8, 11). As a result of the progress made by research studies worldwide, there is now evidence of a central role for DCs in initiating antigen-specific immunity and tolerance, which has been widely translated into different approaches for vaccine design in preclinical and clinical programs (12–15).

**Table 1:** Human DC subsets.

**DC subtype**	**Main surface markers**	**Main PRRs**	**Presence *in vivo***	**Main functions**	**Specific mediators produced upon activation**	**T cell priming ability**	**The role in cancer immunotherapy**	**The role in autoimmune diseases**
pDC	CD11c^−^; CD123^+^; CD303^+^; CD304^+^; CCR2^+^; CXCR3^+^; HLA-DR^low^	STING; TLR7; TLR9; CLEC12A	Resident in lymphoid tissues; also present in tonsils	Antiviral immunity	Type I and III IFN secretion	Poor priming of naive T cells; present and cross-present peptides only after activation	Negatively correlate with prognosis in cancer	Implicated in progression of autoimmune diseases by increased IFNα production and decreased ability to prime Treg cells
cDC1	CD11c^low^; HLA-DR^+^; DEC205^+^; XCR1^+^	STING; TLR1; TRL3; TLR6; TRL8; TLR10; CLEC12A	Resident in lymphoid tissues and also present in blood, peripheral tissues, and lymph nodes	CD8^+^ T cell and T_H_1 priming Cross presentation	Not well-defined	Efficient processing and cross-presentation of exogenous antigens on MHC class I molecules to activate CD8^+^ T cells and prime T_H_1 response	*Cellular immunity against tumor cells and correlates with beneficial prognosis in cancer *Produce CXCL9 and CXCL10 in the TME to promote the recruitment of CD8^+^ T cells into the TME	Implicated in progression of autoimmune diseases by increased production of pro-inflammatory cytokines and T cell activation
cDC2	CD11c^+^; HLA-DR^+^; C11b^+^; CD172a^+^	STING; TLR1-9; CLEC4A; CLEC6A; CLEC7A; CLEC10A; CLEC12A	Resident in lymphoid tissues and also present in blood, peripheral tissues, and lymph nodes	CD4^+^ T cell priming; T_H_17 activation; T_H_1, T_H_2 response induction; Tregs activation	IL-6 and IL-23	Present peptides on MHC class II molecules to CD4^+^ T cells	Inducing CD4^+^ T cell-mediated immunity in cancer	
Langerhans cells	Langerin; Epcam; BDCA1^+^; CD1a^+^; CD11c^High^		Resident in epidermis	Tolerance and priming of immune response	Not well-defined	Not well-defined	Not well-defined	Not well-defined
MoDCs	CD11c^+^; CD11b^+^; HLA-DR^+^; CD1c^+^; CD206^+^; CD209^+^; CD1a^+^; CD172a^+^; CCR2^+^		Differentiate from monocytes in peripheral tissues on inflammation	Inflammation	TNF and iNOS	Induce context dependent differentiation of CD4^+^ T cells into T_H_1, T_H_2 or T_H_17 cells	Mostly studied and used in *ex vivo* generated immunotherapy protocols	Mostly studied and used in *ex vivo* generated immunotherapy protocols

*Overview of key characteristics of the predominant human dendritic cells (DC) subsets: pDCs, plasmacytoid DCs; cDC1s, conventional type I DCs; cDC2s, conventional type 2 DCs; MoDCs, Langerhans cells and monocyte-derived DCs*.

*CCR, chemokine receptor; CXCR3, CXC-chemokine receptor 3; PRR, pattern recognition receptor; T_H_1 cell, type 1 CD4^+^ T helper cell; T_H_2 cell, type 2 CD4^+^ T helper cells; T_H_17 cell, IL-17-producing CD4^+^ T helper cell; TLR, Toll-like receptor; Treg cell, regulatory CD4^+^ T cells. References are provided throughout the main text*.

## DCs Subsets

DCs comprise two major classes: plasmacytoid DCs (pDCs) and conventional or classical DCs (cDC) (Table 1) (11, 16). pDCs represent a small subset of DCs which accumulate mainly in the blood and lymphoid tissues and enter the lymph nodes through the blood circulation. For maturation, pDCs selectively express activating FcR as well as Toll-like receptor 7 and 9 (TLR7 and TLR9). On the contrary, they express low levels of MHC class II and costimulatory molecules in the steady state. Upon recognition of foreign nucleic acids, they start to produce type I interferon (1, 11). pDC-derived IFNα can also induce the activation of other DC subsets or B cells into plasma cells via cytokines and surface signaling (17). cDCs form a small subset of tissue hematopoietic cells present in most of lymphoid and non-lymphoid tissues, where they constantly acquire tissue and blood antigens. cDCs excel in priming naïve T cells due to their superior ability to migrate loaded with antigens to T cell one of lymph node and to process and present antigens. Moreover, cDC1 have a unique potential to induce cellular immunity against intracellular pathogens and malignant cells due to the processing and cross-presentation of exogenous antigens on MHC class I molecules to activate CD8^+^ T cells and T_H_1 cells. On contrary, cDC2 are known potent inducers of CD4^+^ T cell response (1, 11). MoDCs mainly differentiate from monocytes in peripheral tissues during inflammation and induce context dependent differentiation of CD4^+^ T cells into T helper 1 (T_H_1), T helper 2 (T_H_2) or T helper 17 (T_H_17) cells (7).

## DC Activation

**Figure 1:**
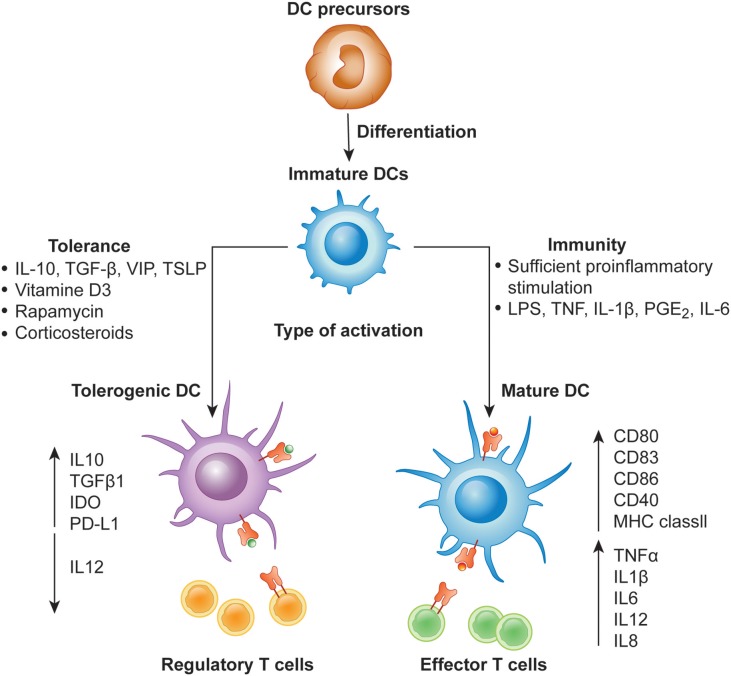
Differentiation of monocyte-derived activated vs. tolerogenic dendritic cells. Dendritic cells (DC) differentiate from DC precursors into immature DCs (iDCs) in the presence of IL-4 and GM-CSF. In the presence of a maturation signal (proinflammatory cytokines and Toll-like receptor ligands), DCs become activated and transition to a stimulatory phenotype, which subsequently leads to the induction of effector/cytotoxic T cell responses. In contrast, incubation of iDCs with different mediators or genetic modification of DCs in the absence of maturation factors can lead to the generation of tolerogenic DCs, which induce anergy, apoptosis or activation of Tregs.

**Figure 2:**
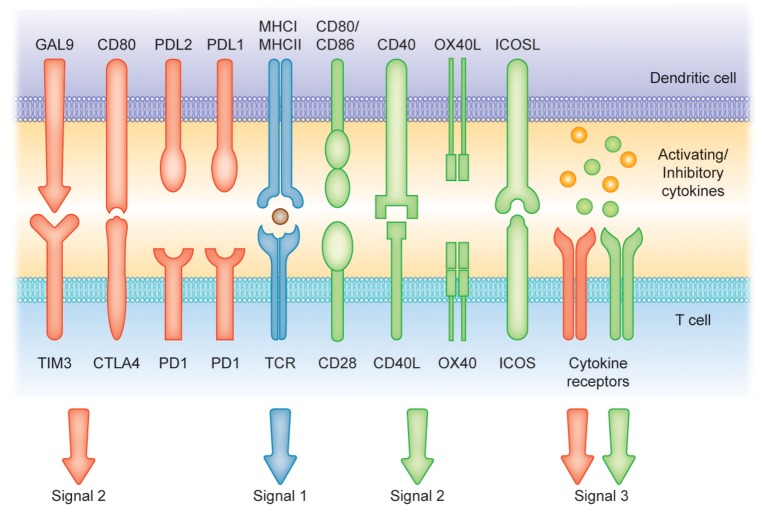
Induction of T cell-mediated immunity or tolerance by DCs. Signal (1) Antigen presentation. Dendritic cells (DCs) can present antigens on MHC I and MHC II molecules to mediate T cell activity. Signals (2) and (3) Costimulatory molecules [belonging to the B7 and tumor necrosis factor (TNF) protein families] and soluble cytokines can provide positive signaling (green arrows and receptors) to prime T cell response. Conversely, CTLA4, cytotoxic T lymphocyte antigen 4; PD1, programmed cell death protein 1; PD-L1, programmed cell death 1 ligand 1 and TIM-3, T cell immunoglobulin and mucin-domain containing-3 and soluble factors such as IL-10 can represent suppressors of T cell activation (red arrows and receptors).

DCs in the resting state are considered to be immature but primed to acquire pathogen-associated molecular patterns (PAMPs) and damage-associated molecular patterns (DAMPs) *in situ* through a variety of surface and intracellular receptors, namely (1) cell surface C-type lectins, (2) surface and intracellular TLRs, and (3) intracellular helicases that recognize nucleic acids, such as retinoic acid-inducible gene I (RIGI) (18) (Table 1). iDCs are potentially tolerogenic due to their capacity to facilitate the suppression of autoreactive T cells and the clonal expansion of Tregs, which might be addressed in the manufacturing of DC-based vaccines for autoimmune disease treatment (19) (Figure 1). DCs undergo a series of phenotypic and functional changes upon exposure to activation signals, leading to their maturation (10). This process is associated with the following events: (1) downregulated antigen-capture activity, (2) increased expression of surface MHC class II molecules and enhanced antigen processing and presentation, (3) increased levels of chemokine receptors, e.g., CCR7, which allows migration of the DC to lymphoid tissues; (4) increased expression of costimulatory molecules associated with the capacity to stimulate or suppress T cells through different signaling axes: CD80/CD86-CD28, CD40-CD40L, OX40L-OX40, ICOSL-ICOS and galectin (GAL)9-TIM3, CD80-CTLA4, PDL1-PD1, PDL2-PD1, respectively (Figure 2); and (5) enhanced secretion of cytokines and chemokines, leading to the development of an immune response T cell subtypes, e.g., CD4^+^ T cells such as T_H_1, T_H_2 and Tregs (8, 20) (Figure 1).

## Induction of T Cell Tolerance vs. Activation by DCs

Different DCs subsets are specialized to capture and process antigens that are presented on MHC molecules and recognized by T cells, resulting in final clonal T cell selection leading to a wide T cell repertoire as summarized in Table 1 (21). Among DC subsets, pDCs show relatively limited priming of naïve T cells, unless stimulated to induce CD8^+^ T cells (22). Conversely, cDC1 provide efficient processing and cross-presentation of exogenous antigens on MHC I molecules to activate CD8^+^ T cells and T_H_1 cell responses as a response to tumor cells or intracellular pathogens (23, 24) and cDC2 are known to be inducers of CD4^+^ T cell responses (25, 26). Importantly, MoDCs can be generated to promote context-dependent differentiation of CD4^+^ T cells toward a T_H_1, T_H_2, or T_H_17 phenotype (27). This variety of T cells represents an infinite tool for specific therapies that increase or decrease T-cell function. The efficient activation of naïve T cells requires the following: (1) binding of the TCR to the peptide-MHC complex on DCs, (2) the interaction of costimulatory molecules at the interface between DCs and T cells, and (3) additional signals from the local environment (28). The presence of these three signals is crucial for full T cell activation (Figure 2). Under inflammatory conditions, large numbers of mature DCs accumulate in T cell areas of the draining lymph nodes for a sustained period of time (29). Mature DCs presenting high levels of antigen/MHC complexes allow strong and sustained TCR occupancy, delivering T cells the main stimulatory signal (30). Simultaneously, high levels of costimulatory and adhesion molecules expressed on mature DCs are required for amplification of the signal initiated by the TCR and for increased adhesion between the DC and the T cell, thus increasing the strength and duration of the interaction, respectively (10). Subsequent strong activation of signaling pathways downstream of the TCR and the costimulatory receptors in the presence of cytokines or factors eliciting immunostimulation and the effector T cell phenotype results in full T cell activation, proliferation, and differentiation into effector and memory cells (Figures 1, 2) (31).

In contrast, DCs that engulf the antigen in the absence of a local inflammatory signal remain in the immature, tolerogenic state with low expression of MHC molecules and costimulatory molecules, such as CD80 and CD86 (9, 32, 33). Presentation of antigen to T cells in the absence of sufficient CD80/CD86 stimulation of CD28 molecules on T cells leads to the activation of anergy-associated genes under the control of nuclear factor of activated T cells (NFAT) and induction of T cell anergy (34, 35) (Figures 1, 2). Moreover, low or no signal through the CD28 receptor is a prerequisite for the induction of Treg differentiation (36). Thus, tolerogenic DCs (tolDCs), DCs with regulatory properties, play a pivotal role in immune tolerance (37).

The tolDC population consists of naïve iDCs or alternatively activated semimature DCs induced by apoptotic cells or regulatory cytokine milieu, such as IL-10 and transforming growth factor β (TGF-β) (20). Immunosuppressive DCs can also be generated under tumor microenvironment-derived factors, such as β-catenin, indoleamine 2,3-dioxygenase (IDO), endoplasmic reticulum (ER) stress, lactate, vascular endothelial growth factor (VEGF), IL-10, TGF-β, prostaglandins, accumulation of adenosine, increased levels of lactate and hypoxia (38–42). TolDCs contribute significantly to the induction and maintenance of immune tolerance through various mechanisms. They promote effector T cell anergy and elimination of autoreactive T cells, participate in the generation and maintenance of a population of naturally occurring Tregs, allow the generation of IL-10-producing T_H_1 and T_H_3 regulatory cells, and allow the conversion of differentiated T_H_1 cells into T_H_2 cells (43, 44). These processes are mainly due to the high production of the regulatory cytokine IL-10, which promotes the generation of Tregs and T_H_2 cells and inhibits DC maturation in a paracrine manner (45). Furthermore, regulatory DCs express various immunomodulatory molecules and immunosuppressive molecules that inhibit proinflammatory immune responses and induce immune tolerance. Indeed, the expression of PD-L, ICOS-L, thrombospondin, prostaglandins, and adenosine was documented to participate in the induction of T cell anergy. A number of mechanisms contribute to the clonal deletion of T cells including the interaction between FasL on DCs and Fas molecules on T cells, the expression of GAL-3 that binds to TIM3 on T cells or the production of IDO that leads to subsequent tryptophan depletion. TolDCs were also reported to induce Tregs or B regulatory cells (Bregs) by the expression of PD-L molecules, Ig-like inhibitory receptors IL-T3 and IL-T4, human leukocyte antigen G (HLA-G), anti-inflammatory cytokines IL-10, TGF-β, IL-27 and IL-35, retinoic acid, heme-oxygenase and IDO (9, 46). Finally, the functionality of tolDCs is connected with their metabolic activity, such as lipid accumulation, enhanced oxidative phosphorylation, fatty acid oxidation, and modulation of glycolysis (39, 47).

## The Role of DCs in Cancer

The immune system plays a critical role in the control of tumorigenesis based on experimental and clinical observations in both mice and humans, as formulated by the cancer immunosurveillance and immunoediting hypothesis (48). The plasticity of malignant cells resulting from their genetic instability may eventually give rise to new phenotypes with reduced immunogenicity and various mechanisms for the evasion of tumor cells from immunosurveillance, leading to malignant proliferation (49). Malignant cells escape immunosurveillance by different mechanisms, some of which are: (1) reduced immune recognition (including loss of tumor antigen expression and MHC class I and costimulatory molecule expression), (2) increased resistance to apoptosis (through STAT3 signaling), or (3) development of an immunosuppressive tumor microenvironment (including the production of cytokines, e.g., VEGF, TGF-β, IL-10 and increased expression of immunoregulatory molecules, e.g., PD-1/PD-L1, TIM-3, LAG-3), which lead to the development of malignant diseases (48, 50). Different DC subsets can be found in the majority of human tumors and play a crucial role in cancer immunosurveillance, as tumor-infiltrating DCs can migrate to regional lymph nodes to present tumor antigens to naïve tumor-specific T cells (51). However, naïve antigen-specific CD8^+^ T cells cannot directly eliminate malignant cells. Thus, to become effector cytotoxic T cells, they need to be activated by professional APCs. Cross-presentation is an essential mechanism that allows DCs to present exogenous antigens on MHC I molecules to CD8^+^ T cells, which become the main mediators of anti-tumor immunity (52). Importantly, the contribution of the different DCs subtypes to cross-presentation a cross-priming (in induction of effector CD8^+^ T cells *in vivo*) varies depending on the experimental setting. cDC1 are mainly associated with superior cross-presentation of tumor antigens to CD8^+^ T cells and polarization of CD4^+^ T cells into T_H_1 phenotype resulting in induction of anti-tumor immunity (53–55). cDC2 and MoDCs may also cross-present tumor antigens and cDC2 are known to be essential for priming of anti-tumor CD4^+^ T cell response (56). Moreover, the effector activity of T cells depends on DC-derived cytokines, including IL-12 and type-I IFN. Both cDC1s and cDC2s produce IL-12 following TLR stimulation. Tumor infiltrating cDC1s are also the main producers of different chemokines, including CXCL9 and CXCL10, which help to promote the recruitment of CD8^+^ T cells into the tumor microenvironment (TME) (57). Therefore, elevated levels of tumor-infiltrating DCs inversely correlate with tumor grade and stage and have a robust prognostic value in multiple cancers, including non-small-cell lung carcinoma (NSCLC), melanoma, renal cell carcinoma, breast cancer, ovarian, and colorectal carcinoma (58–63).

However, the tumor microenvironment employs various mechanisms that lead to the functional impairment of DCs (7). First, in the TME, iDCs differentiate from hematopoietic progenitors following an encounter with an antigen/danger signal (64). However, the differentiation of DCs in the TME is often mediated by the interplay between IL-6 and macrophage colony-stimulating factor (M-CSF), resulting in the recruitment and accumulation of functionally deficient and frequently iDCs unable to induce the proliferation of tumor-specific CD4^+^ and CD8^+^ T cells (65, 66). Second, DCs in their function as APCs are sampling tumor antigens through the capture of dying tumor cells and initiating the anti-tumor immune response. Dying tumor cells provide three different signals to DCs and other phagocytes: “find-me,” “eat-me “and “do not eat me” (67). A number of find-me signals have been characterized that act in a context-dependent manner, including lipid lysophosphatidylcholine (LPC), sphingosine 1-phosphate (S1P), CX3CL1 and the nucleotides adenosine triphosphate (ATP) and uridine triphosphate (UTP) (67). Immunogenic phagocytosis is mediated by eat-me signals, namely, ecto-calreticulin (CALR), surface-heat shock protein (HSP) 90, and phosphatidylserine (68, 69). The “do not eat me” signals serve as negative regulators of phagocytosis, mainly including CD47 and lactoferrin (70). Therefore, homeostatic clearance of dying cancer cells could be accelerated or impaired by the different molecules provided by tumor cells, which results in enhanced or impaired phagocytosis of malignant cells (71). Third, the functional capacity of DCs in the TME is negatively impacted through different mechanisms, including the activation of STAT3 signaling in DCs via different cytokines frequently expressed in tumors (IL-6, VEGF and IL-10) (72). Moreover, tumors may condition local DCs to form suppressive T cells, such as Tregs, IL-13-producing CD4^+^ T cells and natural killer T cells (NKT cells), leading to a tumor-induced functional deficiency of DCs that results in decreased expression of costimulatory molecules, decreased production of IL-12, suppressed endocytic activity, inhibited antigen-processing machinery, and poor viability (73–77). Altogether, these and other findings suggest that malignant cells can exploit DCs to evade immunity. However, the majority of clinical protocols harnessing patient DCs do not consider the fact that DCs once administered back to patients might quickly lose their activity.

## The Role of DCs in Autoimmunity

Previous studies have described the link between peptide presentation by HLA class II molecules expressed on APCs and autoimmune diseases. In different autoimmune diseases, DCs are bearing certain autoimmune risk-conferring HLA class II molecules with the distinct hotspots in the peptide-binding groove that favor the presentation of particular self-antigens that will ultimately be recognized by self-reactive TCR. In the case of type 1 diabetes (DM1), the presence of specific amino acid in the binding groove of HLA-DQ8 alleles favors the binding of insulin-derived peptides. Similarly, in the case of rheumatoid arthritis (RA), HLA-DR4 molecules bearing the conserved amino acid motif (shared epitope) favor the presentation of citrullinated self-peptides leading to activation of citrulline-specific CD4^+^ T cells and subsequent production of anti-citrulline antibodies that foster RA but prevent natural ligands bearing arginine instead of citrulline (78).

Aberrant cDC and pDC phenotypes and functions due to underlying genetic defects or a chronic inflammatory environment were shown to be associated with the development of various autoimmune diseases, such as RA, systemic lupus erythematosus (SLE), multiple sclerosis (MS) or DM1 (45, 79–81). DCs can either induce or suppress the autoreactive T cell response, and their effect depends on the DC subset, the degree of maturity, signals obtained from the local microenvironment and crosstalk with other immune and stroma cells. Under non-inflammatory conditions, lymphoid-resident immature cDCs or specialized types of tolDCs bearing self-antigens suboptimally activate naïve CD4^+^ and CD8^+^ T cells, thus maintaining immune tolerance and affecting the regulation of autoimmune diseases. Aberrant intrinsic tolDC function, such as impaired IL-10 secretion, defective ability to remove apoptotic cells, defective antigen processing machinery or absent negative regulators of inflammation, can contribute to DC hyperactivation and trigger autoimmunity (82–85). DC hyperactivation might also result from environmental triggers such as an inflammatory cytokine milieu induced by bacteria (86, 87), excessive IFN production in response to viral infection as observed in DM1 (88), oxidative stress induced by noxious agents as observed in RA (89) or danger signals released under cell stress or from necrotic and late apoptotic cells as documented in SLE and DM1 (90, 91). Activated cDCs accumulate in lymphoid and non-lymphoid tissues during autoimmune disease progression. Hyperactivated cDCs present self-antigens, prime naïve autoreactive CD4^+^ T cells including follicular helper T cells, promote cross-priming of CD8^+^ T cells and orchestrate the maturation of B cells leading to the subsequent expansion of autoantibodies and immune complex formation (81). Furthermore, mature cDCs generate an inflammatory environment by producing high levels of pro-inflammatory cytokines such as IL-1β, IL-6, IL-12, and IL-23 that induce a deleterious imbalance between T_H_1, T_H_2, and T_H_17 cells and contribute to local inflammation and tissue destruction. Although partially regulated, the autoimmune response persists due to ongoing stimulation of autoreactive T cell clones and B cell clones. pDCs play a central role in the pathogenesis of IFN-driven autoimmune diseases such as SLE and psoriasis. In SLE, pDCs are activated by immune complexes formed by the aggregation of autoantibodies, stress proteins, such as high mobility group box 1 (HMGB1), and self-DNA released from apoptotic cells that have not been cleared or by nucleic acid-containing nets released from activated neutrophils. These complexes are delivered to endolysosomes to activate TLR7 or intracellular DNA sensors, such as cGAS-STING, to further activate pDCs and IFN-α secretion (92–94). On the other hand, pDCs can also reduce autoimmune responses by secreting IDO and inducing Tregs depending on the disease stage and signals from local tissues (95, 96).

## DC-based Cancer Immunotherapy

Immunotherapy strategies harnessing DCs have been developed based on their unique capacity to coordinate innate and adaptive immune responses (10). The main aim of DC-based cancer vaccination is to induce tumor-specific cellular and humoral immunity resulting in the reduction of tumor mass and induction of immunological memory, which will control cancer relapse. Therefore, a critical step in cancer vaccine preparation is to provide mature DCs with specific tumor antigens. This can be achieved by the following: (1) culturing *ex vivo* DCs derived from patients with tumor antigens and activation stimuli and subsequently transferring the activated DCs back into patients or (2) inducing tumor antigen uptake by DCs directly *in vivo* (7, 97) The first proof-of-principle studies exploring DC immunotherapy were performed in the early 1990s based on the discovery that DCs can be obtained from CD14^+^ monocytes or CD34^+^ progenitors from leukapheresis products by culturing the cells *in vitro* in the presence of IL-4 and GM-CSF for 5–6 days (98). The first clinical study of a DC anti-cancer vaccine in B-cell lymphoma patients was reported by Hsu and colleagues in Nature Medicine in 1996 (99). Since then, ~200 clinical studies have been performed of single treatments using mostly monocyte-derived DCs and measuring the immune response, which have been comprehensively reviewed elsewhere (12, 13, 97, 100). These studies concluded that DC-based vaccines are safe and potent for inducing the expansion of circulating tumor-specific CD4^+^ and CD8^+^ T cells (101–103). Although an anti-tumor immune response is frequently observed, objective clinical responses remain low, with a classic objective tumor response rate rarely exceeding 15%, as currently concluded in the meta-analysis provided by Anguille and colleagues (13, 14, 21). Although considerable progress has been made over the years, most of the studies have, unfortunately, been performed in late-stage patients with strong immunosuppression mechanisms already in place (104–106). To date, limited phase II and III trials (Table 2) have been performed with DC-based immunotherapy and, therefore, more clinical studies evaluating early stage patients or patients with preneoplasia are strongly needed.

**Table 2:** Phase II and III clinical trials currently testing the therapeutic efficacy of dendritic cell-based anticancer immunotherapy.

**Cancer type**	**Trial phase**	**Type of vaccine**	**Status**	**Intervention**	**ClinicalTrial.gov identifier**
Breast cancer	II	Autologous DC-CIK combinations	Active, not yet recruiting	CIKs, Capecitabine	NCT02491697
CRC	III	Autologous DCs loaded with tumor cell lysate	Active, not yet recruiting	DCs+FOLFOX6 (Oxaliplatin, 5-Fluorouracil)	NCT02503150
Follicular lymphoma	II	Autologous DCs	Active, recruiting	Intranodal DCs+pembrolizumab	NCT02677155
GBM	II	Autologous DCs loaded with tumor cell lysate	Active, not yet recruiting	Tetanus Diphteria toxoid, Basiliximab	NCT02366728
	II	Autologous DCs loaded with tumor cell lysate	Active, not yet recruiting	Nivolumab	NCT03014804
	II	Autologous DCs	Active, recruiting	Tetanus Diphteria toxoid, GM-CSF	NCT02465268
Melanoma	II	Autologous DCs loaded with tumor cell lysate	Active, recruiting	DCs+IL-2	NCT02718391
	II	Autologous DCs loaded with tumor cell lysate	Active, recruiting		NCT02301611
	II	Autologous DCs loaded with TAAs	Active, not yet recruiting	Hiltonol	NCT02334735
	III	Autologous DCs loaded with TAAs	Active, recruiting		NCT02993315
	III	Autologous DCs + irradiated autologous tumor cells	Terminated		NCT01875653
Multiple myeloma	II	Dendritomas	Active, not yet recruiting	Autologous stem cell transplant with Melphalan, lenalidomide	NCT02728102
NSCLC	II	Autologous DCs + HHP-treated tumor cells	Completed	DCs+carboplatin, paclitaxel	NCT02470468
Ovarian cancer	II	Autologous DCs + HHP-treated tumor cells	Active, recruiting	DCs+carboplatin, paclitaxel	NCT02107937
	II	Autologous DCs + HHP-treated tumor cells	Completed	DCs+carboplatin, paclitaxel	NCT02107950
Prostate	II	Autologous DCs loaded with TAAs	Active, recruiting		NCT02362451
	II	Autologous DCs loaded with TAAs	active, not yet recruiting		NCT02692976
	II	Autologous DCs + HHP-treated tumor cells	Completed	DCs+docetaxel	NCT02105675
	II	Autologous DCs + HHP-treated tumor cells	Completed	DCs+standart of care hormone therapy (Leuprolid, Goserelin)	NCT02107391
	II	Autologous DCs + HHP-treated tumor cells	Completed		NCT02107404
	II	Autologous DCs + HHP-treated tumor cells	Completed	DCs+standart radiotherapy	NCT02107430
	III	Autologous DCs + HHP-treated tumor cells	Active, recruiting	DCs+docetaxel, taxotere	NCT02111577
RCC	II	Allogeneic DCs (Intuvax)	Active, not yet recruiting	DCs+Sunitinib	NCT02432846
	II	Autologous DCs loaded with tumor cell lysate	Active, not yet recruiting	DCs+CIKs	NCT02487550
	III	Autologous DCs	Terminated	DCs+Sunitinib	NCT01582672
Uveal melanoma	III	Autologous DCs + autologous tumor RNA	Active, recruiting	DCs+adjuvant	NCT01983748

*CIK, cytokine-induced killer; CRC, colorectal carcinoma; DC, dendritic cell; GMB, glioblastoma multiforme; GM-CSF, granulocyte macrophage colony-stimulating factor; HHP, high hydrostatic pressure; IL-2, interleukin 2; NSCLC, non-small cell lung carcinoma; RCC, renal carcinoma; TAA, tumor associated antigen*.

## *Ex-vivo* DC-based Vaccines

Different *ex vivo* DC-based immunotherapy clinical trials have recently been concluded with encouraging clinical outcomes (100). Completed clinical studies have analyzed the following: (1) different protocols for DC preparation, (2) different DC activation stimuli, (3) different forms of antigen preparations from short peptides to complex whole-tumor-cell hybrids, and (4) different types of DC vaccine applications. First, the FDA-approved cell-based therapy for the treatment of hormone-refractory prostate cancer Provenge (Sipuleucel-T) is a vaccine consisting of autologous peripheral blood mononuclear cells (PBMCs) obtained by leukapheresis, including DCs activated with a fusion protein of a prostate antigen (prostatic acid phosphatase; PAP) and GM-CSF. Treatment with Sipuleucel-T resulted in a 4.1-month-prolonged median survival compared with placebo (25.8 vs. 21.7 months). The impact of this first FDA-approved cancer vaccine has been significant, however this product is not readily available for different reasons, including logistic and financial problems (107). More phase II and III clinical trials using autologous MoDCs obtained from patient-derived CD14^+^ blood monocytes or from the CD34^+^ progenitors are shown to be effective against different cancer types and are summarized in Table 2. Phase III clinical trials using Mo-DC-based cancer vaccination are ongoing in metastatic colorectal cancer (NCT02503150, autologous tumor lysate), castration-resistant prostate cancer, which is combined with first-line chemotherapy (NCT02111577; VIABLE, MoDC vaccine loaded with antigens from an allogeneic apoptotic tumor cell line) and melanoma (NCT01983748, autologous tumor RNA antigen). In addition to colorectal, prostate cancer and melanoma cancer, DCs are intensively studied in glioma and renal and ovarian carcinoma (Table 2) (108, 109).

## *In vivo* DC Targeting

Another approach to recruit natural DCs for cancer immunotherapy is to target DC subsets *in vivo* via specific receptors, e.g., DEC205, CLEC9A, and langerin to target cDC1s; CLEC4A4 to target cDC2; CLEC7A (dectin 1) to target cDC2 and MoDCs; CD209 (DC-SIGN), mannose receptor and macrophage galactose-type lectin to target macrophages, using antibodies to deliver antigens and activating agents (110–112). Compared to *ex vivo* DC generation protocols, *in vivo* targeting allows vaccines to be produced on a larger scale and, most importantly, allows direct activation of natural DC subsets in the patient's body. Importantly, in the absence of adjuvants, targeting antigens to DCs might induce tolerance rather than anti-tumor immunity, which would have substantial value in the context of autoimmunity. Currently, numerous *in vitro* and *in vivo* studies in humans are focused on DC-targeting vaccine development. In a phase I trial, a DC-based vaccine consisting of a fully human anti-DEC205 monoclonal antibody fused to the tumor antigen NY-ESO-1 and accompanied by a topical or subcutaneous application of TLR agonists (resiquimod) showed the efficient generation of NY-ESO-1-specific cellular and humoral responses and led to partial clinical responses without toxicity (113). Nevertheless, the correlation with clinical responses remains unclear, and larger studies will be needed to evaluate the efficacy of this therapy. Clinical trials of anti-DEC205-NY-ESO-1 are currently ongoing in acute myeloid leukemia (NCT01834248), ovarian cancer (NCT02166905) and melanoma (NCT02129075). The advantage of such an approach is that maturation stimuli activate only DCs targeted by the antibodies, thereby preventing any toxicity or undesirable systemic activation (13).

A different approach of targeting DCs *in vivo*, called GVAX, involved engineering irradiated gene-transfected tumor cells to secrete GM-CSF to stimulate the recruitment and activation of APCs (114). One phase II trial testing an allogeneic pancreatic cell line that secretes GM-CSF in combination with/without recombinant live attenuated *L. monocytogenes* engineered to secrete mesothelin (CRS-207) and low dose cyclophosphamide resulted in the recruitment of T cells into the TME and improved overall survival in patients with advanced pancreatic cancer (115, 116). However, a phase IIB study failed to show improved overall survival in patients treated with the combination or CRS-207 alone compared with the survival of patients on chemotherapy. Importantly, two different phase III clinical trials to evaluate the therapeutic efficacy of GVAX in prostate cancer patients were conducted. The VITAL-1 trial comparing GVAX to docetaxel plus prednisone in castration-resistant prostate cancer was terminated after showing low efficacy by interim analysis. VITAL-2 comparing GVAX in combination with docetaxel vs. docetaxel in combination with prednisone was also terminated based on interim results showing an increased risk of death in the GVAX arm compared to the control group (117). In this line, promising results showing that FMS-like tyrosine kinase 3 ligand (FLT3L) administration enhanced anti-tumor immunity and limited the tumor cell growth in mouse models (118), are currently followed by clinical trials (NCT01811992, NCT01976585, NCT02129075, and NCT02839265).

## DC-based Therapy of Autoimmune Diseases

The current treatment of most autoimmune diseases involves lifelong administration of systemic immunosuppression drugs coupled to anti-inflammatory therapies and hormone replacement. In addition, systemic immunosuppression is inevitably associated with undesirable side effects. Thus, the main goal of autoimmune disease treatment would be the long-term reinduction of self-tolerance. With respect to autoimmune disorders, cell therapy based on autologous tolDCs generated from peripheral blood monocytes following *ex vivo* generation in GM-CSF and IL-4 cell culture medium might be beneficial over standard immunosuppressive treatment in terms of its complex effect on the immune system and the possibility to restore long-term antigen-specific tolerance while avoiding generalized immunosuppression.

In order to achieve the best *in vivo* tolDC efficacy, all the parameters of tolDC therapy, namely, optimal dose, administration route, and frequency of tolDC administration, have to be properly defined as we believe they could dictate what kinds of immune responses are activated to modulate autoreactive T-cells and induce immune tolerance. To date, the best route of tolDC administration is still not known and several challenges remain to allow tolDCs to migrate into draining lymph nodes for T cell encounter or to reach the site of inflammation. In most clinical trials, tolDCs have been administered subcutaneously or intradermally proximal to the inflammatory site to increase tolDC migration to draining lymph nodes where autoreactive T cells predominate and to reach the site of inflammation (119). At the same time, intranodal application and direct administration into the intestinal lesions of tolDCs has also been tested in phase I clinical trials in patients with MS and Crohn's disease, respectively (120). In MS, however, tolDC shuttle across the blood brain barrier seems to be required for the efficient treatment of MS. Recent data suggest that the introduction of de *novo* CCR5 expression using mRNA electroporation into tolDCs might facilitate migration od tolDCs into the inflamed central nervous system and improve the treatment outcome in MS (121). Moreover, the ability of tolDCs to modulate T cell responses might be influenced by the current clinical status of the patient. Indeed, we documented in our studies that hyperglycemia reduces the ability of tolDCs to induce stable Tregs from naive T lymphocytes that can suppress antigen-specific T-cell anergy (122, 123). In that case, metabolic control, we believe, might be relevant for refining the inclusion criteria for clinical trials involving patients with DM1 and the maintenance of a tight metabolic control seem to be beneficial in patients considered for tolDC therapy.

Similar to DC-based cancer vaccines, a number of *in vivo* studies have documented that tolDCs require pulsing with relevant antigens to reach efficient clinical responsiveness following tolDC therapy (124). However, in some instances, antigen loading tolDCs leads to a worse condition and a higher incidence of autoimmune disease (125, 126). In contrast, different *in vivo* studies have suggested that the presence of autoantigen is not necessary for tolDC preparation as tolDCs may upload relevant autoantigens once injected *in vivo* and induce antigen-specific tolerance (127). Moreover, autoimmune diseases are not commonly defined by one universal autoantigen. Suitable disease-specific autoantigens such as insulin and glutamic acid decarboxylase 65 (GAD65) or transgenic myelin oligodendrocyte glycoprotein (MOG) or myelin basic protein have been defined in DM1 and MS, respectively (128, 129). However, in some autoimmune disorders, the specific autoantigen remains unidentified despite significant effort. In addition, not all patients display a uniform autoantigen pattern as antigen spreading, posttranslational modification, and development of neoantigens usually occur during the progression of the disease and complicate the search for the target antigens of the autoimmune response (128). A possible strategy seems to be to use a surrogate “universal” antigen, e.g., HSPs that are ubiquitously expressed in different types of inflammatory tissues (130).

## *Ex-vivo* DC-based Vaccines

*The ex vivo* generation of stable, maturation-resistant tolDCs followed by their adoptive transfer represents novel immunotherapy for the antigen-specific treatment of autoimmune disorders. TolDCs can be established from monocytes from a patient's blood cultured using various pharmacological agents Vitamin D (VitD) and its analogs, dexamethasone, rapamycin, salicylates, and NF-κB inhibitors, a cocktail of immunomodulatory cytokines (IL-10, TGF-β), growth factors (GM-CSF, M-CSF), and pathogen products and with the use of apoptotic cells or genetic engineering (131). All of these approaches generally suppress the maturation or activation of DCs and reduce the ability of DCs to produce IL-12p70 through different mechanisms (131). Additional activation of tolDCs by lipopolysaccharide (LPS) or its non-toxic analog monophosphoryl lipid A (MPLA) has been shown to improve the antigen-presenting capacity and migratory ability of tolDCs (132). Common features of tolDCs include low antigen presentation capacity combined with the loss or reduction of costimulatory signals, expression of inhibitory molecules, and an anti-inflammatory cytokine profile. Generated tolDCs can be loaded with one or more antigens to confer specificity. To do so, suitable disease-associated antigens such as preproinsulin peptides or GAD65 for DM1, basic myelin proteins for MS or thyreoglobulin for autoimmune thyroiditis are necessary. Once injected *in vivo*, tolDCs are expected to induce antigen-specific tolerance through various mechanisms, such as induction of autoreactive T cell anergy, induction of apoptosis, and induction of various types of Tregs and Bregs (133).

The first clinical study on tolDC therapy was conducted in 2011 in adult patients suffering from autoimmune DM1. TolDC therapy was safe, and some patients exhibited increased blood levels of B220^+^CD11c^+^ B cells together with evidence for C-peptide reactivation posttreatment (134). To date, further phase I/II clinical studies have been completed or are currently in progress in DM1, RA, MS, and Crohn's disease (Table 3) (135). A Rheumavax study on tolDCs from RA patients established by NF-κB inhibitor and pulsed with citrullinated peptides documented decreased numbers of effector T cells, decreased levels of proinflammatory cytokines and chemokines and reduced DAS 28 score (Table 3) (136). Another study tested the safety, feasibility, and acceptability of dex-VitD3-treated tolDCs pulsed with autologous synovial fluid as a source of autoantigens (AutoDecRa study) or tolDCs generated in the presence of TNF-α and relevant disease peptides (CreaVax study) in patients with RA. Both studies indicated tolDC therapy to be safe and showed signs of clinical improvement (137). Intraperitoneal administration of Dex/VitD-treated tolDCs in Crohn's disease revealed clinical improvement in some patients associated with an increase in Tregs and reduction in IFN-γ levels (138). Recently, Zubizarreta and colleagues reported the safety, feasibility, and signs of efficacy of tolDC therapy in patients suffering from MS and neuromyelitis optica. Indeed, i.v. administration of peptide-loaded tolDCs led to a significant increase in the production of IL-10 in PBMCs stimulated with the peptides as well as an increase in the frequency of regulatory IL-10-producing Tregs (139, 140). Additionally, follow-up studies testing the safety of VitD3 or dexamethasone-treated tolDCs loaded with relevant disease peptides are currently recruiting patients with MS (135).

**Table 3:** Clinical trials currently testing the therapeutic efficacy of dendritic cell-based immunotherapy in autoimmune disorders.

**Disorder**	**Trial phase**	**Vaccine generation**	**Antigen**	**Status**	**ClinicalTrial.gov identifier**
DM1	I	Antisense oligonucleotides against CD40, CD80 and CD86		Completed	NCT00445913
	I	VitD3	Proinsuline peptide	COMPLETED	NTR5542
Rheumatoid arthritis	I	NF-κB inhibitor, Bay 11-7082	Citrullinated peptides of vimentin, collagen type II and fibrinogen α and β chain	Completed	NCT00396812
	I	Dex, VitD_3_, MPLA activated	Autologous synovial fluid	Completed	NCT01352858
Crohn's disease	I	Dex, VitA, activated with IL-1β, IL-6, TNF-α, PGE2		Completed	NA
Multiple sclerosis	I	Dex	Myelin peptides	Completed	NCT02283671
	I	VitD3	Myelin peptides	Active/recruiting	NCT02618902

*Dex, dexamethason; DM1, diabetes mellitus 1; MPLA, lipopolysaccharide analog monophosphoryl lipid A;NF-κB, nuclear factor kappa-light-chain-enhancer of activated B cells; PGE2-prostaglandin E2; TNF-α, tumor necrosis factor alpha; Vit, vitamin*.

## *In vivo* DC Targeting

*Ex vivo*-generated tolDCs have certain disadvantages, such as laborious, patient-specific, tailored-made preparation and high cost. To overcome these limitations, new approaches are being conducted to establish tolDCs *in vivo. One* possibility is the selective antigen-specific targeting of the DC-restricted endocytic receptor DEC205 with monoclonal antibodies in the absence of maturation stimuli to promote immunological tolerance (141). Another approach exploits coadministration of free autoantigens or autoantigens encapsulated with nanoparticles, microparticles, or liposomes bearing tolerogenic factors that are delivered specifically to DCs (142) or infusion of early-stage apoptotic cells that possess immunomodulatory properties and should prevent autoimmunity or even treat ongoing inflammatory processes (143). Another strategy is based on the non-inflammatory natural process of clearance of red blood cells by splenic APCs. Indeed, transfusion of engineered erythrocytes with covalently attached autoantigenic peptides was documented to induce antigen-specific immune tolerance via the uptake and processing of apoptotic cellular carriers for tolerogenic presentation by host splenic APCs in DM1 and SLE (144).

## DCVAC, an Immunotherapy Approach Harnessing DCs to Treat Both Cancer and Autoimmunity

**Figure 3:**
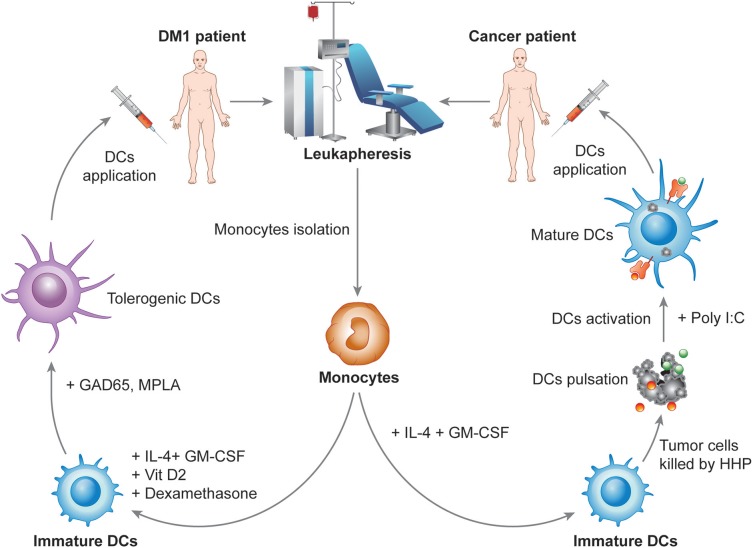
Design of the manufacturing of DCVAC immunotherapy for cancer and autoimmune disorders. In the manufacturing of DCVAC, monocytes are harvested from patient blood by leukapheresis. For cancer patients, monocytes are differentiated into immature DCs (iDCs) in the presence of IL-4 and GM-CSF cytokines for 6 days in a GMP facility. iDCs are subsequently incubated with tumor cell lines treated with high hydrostatic pressure to induce immunogenic cell death of the tumor cells. Finally, DCs are activated using TLR3 ligand polyI:C. Aliquots of DCVAC DC-based immunotherapy are frozen in liquid nitrogen and shipped to the treatment sites. After being thawed and diluted, DCVAC is administered subcutaneously at various treatment intervals depending on the trial design. Similarly, for DM1 patients, monocytes are differentiated into iDCs in the presence of IL-4 and GM-CSF. In contrast, tolerogenic factors (dexamethasone and VitD2) are introduced to the culture at the indicated days to induce the tolerogenic phenotype of DCs. Tolerogenic DCs are finally activated with the lipopolysaccharide analog monophosphoryl lipid A (MPLA), aiming to improve the tolerogenic properties of the DCs.

DCVAC, an investigational immunotherapy treatment based on a new active cellular immunotherapy platform, aims to treat cancer or autoimmune diseases by inducing or suppressing patients DCs, respectively. The unique capacity of DCs to induce both immune activation and tolerance under distinct circumstances is widely used for the preparation of several immunotherapy products currently tested in multiple phase I clinical trials in patients with autoimmune diseases and phase II and III clinical trials in cancer patients. The most advanced immunotherapy treatment in the oncology field is designed for prostate (DCVAC/PCa), ovarian (DCVAC/OvCa) and lung (DCVAC/LuCa) cancer patients. Based on theoretical assumptions and experimental data, cancer immunotherapy has the greatest potential when applied at the early stages of the disease or to patients following a radical surgical intervention after a removal of a large amount of tumor tissue (145). In contrast, in advanced stages of the disease, cancer immunotherapy might have a limited impact on malignant cell eradication due to the establishment of tumor-induced immunosuppression (68, 146). Moreover, preclinical and clinical testing supports the fact that the goal of immunotherapy in the late disease stages is not necessarily complete eradication of the tumor but rather the establishment of an equilibrium state between the host immune system and malignant cells (147). Therefore, it is beneficial to combine immunotherapy with other treatment options, for instance, chemotherapy or radiotherapy (145, 148). The concept of combined chemo-immunotherapy explores the fact that cytostatic treatment might not only eradicate the tumor mass but also neutralize tumor-induced immunosuppression, thus facilitating the effect of the concurrent immunotherapy, as discussed previously in detail elsewhere (146, 149–151). Therefore, numerous phase II clinical trials are ongoing to evaluate the potential of DCVAC in patients at various stages of disease (Table 2). The DCVAC technology in cancer therapy has been focused on a number of principles. First, DCVAC technology using high hydrostatic pressure (HHP)-treated allogenic tumor cell lines is used to activate patients' DCs by a broad range of tumor antigens to induce a complex anti-tumor immune response. The major advantages of this method are that (A) multiple epitopes can be presented on MHC molecules of different haplotypes, thus having the potential to induce both CD4^+^ and CD8^+^ T cell responses to a wide spectrum of antigens and (B) for the time it takes for antigen processing results in prolonged antigen presentation. Second, the concept of combination therapy is also being investigated in patients with advanced cancer in combination with multiple treatment modalities, including chemotherapy and hormone therapy, to produce synergistic effects and to improve the clinical outcome. Third, long-term activation of the immune response is achieved. DCVAC is applied in several doses over a prolonged period, which leads to enhanced stimulation of the anti-tumor immune response in the patient. DCVAC/PCa, DCVAC/OvCa, and DCVAC/LuCa immunotherapy is manufactured from monocytes harvested from patient leukapheresis (Figure 3). Monocytes are differentiated *ex vivo* into iDCs in the presence of IL-4 and GM-CSF for 6 days (152–154). iDCs are subsequently loaded with tumor cell lines of the appropriate origin based on overlap with the expression profiles of tumor-associated antigens (Figure 3) (155, 156). A particular way to enhance the immunogenicity of tumor cells used in the protocol is to induce immunogenic cell death (ICD) and increase the exposure/release of DAMPs to enhance DC maturation. HHP is a potent inducer of ICD, as documented both *in vitro* and *in vivo* (157–162). Moreover, HHP treatment of tumor cells can be easily standardized and performed in good manufacturing practices (GMP) conditions to allow its incorporation into the manufacturing protocol. The patient's own DCs engulf the dying tumor cells and, once activated using TLR3 ligand polyI:C, present tumor antigens on their surface (152). The resulting product is frozen, stored in liquid nitrogen and shipped to the treatment site. The first dose is administered to the patient ~4 weeks after leukapheresis. A single leukapheresis yields up to 15 doses of DCVAC, which is sufficient to treat the patient for more than 1 year. After being thawed and diluted, DCVAC is administered subcutaneously at various treatment intervals, depending on the trial design. After administration, mature DCs migrate to the draining lymph nodes and activate a tumor-specific immune response (163, 164). Similar to boosting the immune system in cancer patients, DCVAC technology might be exploited to regulate unwanted autoimmune processes and induce long-term antigen-specific tolerance in patients suffering from autoimmune disease, such as DM1. DCVAC aimed at the immunotherapy of patients with DM1 consists of tolDCs generated *in vitro* from peripheral monocytes isolated from patient leukapheresis (Figure 3). First, iDCs are generated from monocytes in the presence of GM-CSF and IL-4, similar to DCVAC for cancer patients. Then, in contrast to DCVAC, tolerogenic factors (dexamethasone and VitD2) are introduced to the culture at the indicated days to induce the tolerogenic phenotype of DCs. As antigen loading might decrease the disease-protective effect of tolDCs in animal models of DM1, diabetogenic antigens are not introduced into the manufacturing protocol (125, 165). Finally, tolDCs are activated with the MPLA to improve tolerogenic properties as reported previously (132). Ultimately, tolDCs maintain a semimature phenotype and exhibit tolerogenic properties even under strong inflammatory conditions (166). Overall, DCVAC active cellular immunotherapy represents a personalized treatment of prostate, ovarian, and lung cancers and potentially also autoimmune diseases. The aim of the ongoing phase I to phase III clinical trials is to evaluate the efficacy and confirm the safety of this approach in order to offer new treatments for cancer malignancies and autoimmune disorders.

## Conclusions

DC vaccination has proven to be safe and feasible in multiple clinical trials, as shown over the past two decades. Vaccination strategies involving DCs have been designed with regard to the unique capacity of these cells to coordinate innate and adaptive immune responses. The main aim of DC therapy is therefore to induce tumor-specific effector T cells that can reduce the tumor growth and induce immunological memory to control tumor relapse in cancer patients. In contrast, the main aim of DC therapy in autoimmune disorders is to expand and induce T cells, usually Tregs, that suppress immunity. Significant advances have been achieved in the last 20 years, and DC vaccines are continuously being optimized. The contemporary view on the potential role of DCs in cancer and autoimmune therapy has expanded remarkably, moving from *ex vivo* generated DC-based vaccines to a broad array of therapeutic options. However, we still need to learn more about potential combination therapy which could promote the efficacy of established cancer therapies and the identification of reliable biomarkers that can predict the propensity of cancer patients to benefit from DC-based immunotherapy.

## Author Contributions

All authors listed have made a substantial, direct and intellectual contribution to the work, and approved it for publication.

### Conflict of Interest

JF and LP-J are employees of Sotio; JB and RS are minority shareholders of Sotio.
